# Dissecting the Inter-Substrate Navigation of Migrating Glioblastoma Cells with the Stripe Assay Reveals a Causative Role of ROCK

**DOI:** 10.1007/s12035-013-8429-3

**Published:** 2013-02-24

**Authors:** Sonja Mertsch, Patrick Oellers, Michael Wendling, Werner Stracke, Solon Thanos

**Affiliations:** Institute of Experimental Ophthalmology, School of Medicine, Westfalian-Wilhelms University, Albert Schweitzer Campus 1, Building D15, 48149 Münster, Germany

**Keywords:** Stripe assay, Glioblastoma cells, In vitro, Cell migration, ROCK

## Abstract

A hallmark of gliomas is the growth and migration of cells over long distances within the brain and proliferation within selected niches, indicating that the migrating cells navigate between complex substrates. We demonstrate in the present study a differential preference for migration that depends on Rho-associated coil kinase (ROCK) signaling, using the alternating Bonhoeffer stripe assay. Membrane fractions from nonmyelinated and myelinated brain areas from female rats, purified myelin also from female rats, and commercial extracellular matrix were used as substrates, with each substrate being tested against the others. The human tumor cell lines exhibited a clear preference for extracellular matrix over all other substrates and for myelinated over nonmyelinated tissue. ROCK signaling was different when cells were cultured on either substrate. The ROCK inhibitor Y27632 significantly attenuated and neutralized the preference for extracellular matrix and myelin, indicating that ROCK controls the substrate selectivity. The findings of this study pave the way for navigation-targeted therapeutics.

## Introduction

Glioblastoma multiforme is the most common primary brain tumor, with an incidence of 3–5 per 100,000 population [1]. The prognosis has only slightly improved recently, with a current median survival of 14.6 months today [2, 3]. One of the first hallmarks of glioblastoma is nonresectable diffuse invasion into healthy brain parenchyma, which leads to recurrences in virtually every patient even when surgery and chemo/radiotherapy are applied [2–4]. A second hallmark is the long-distance migration from the primary tumor, often along white matter tracts, indicating a high affinity for substrates constituting white matter [4–6]. Given the densely packed nature of cerebral tissue, it is difficult to distinguish between the different affinities of tumor cells toward particular components such as myelin, neuronal tissue, extracellular matrix (ECM), or capillaries. A migrating glioma cell may simultaneously encounter gray matter, white matter, and ECM and exhibit different affinities to these components.

Commonly used in vitro migration models such as the radial migration assay, wound healing assay, and Boyden chamber assay use components of the ECM as migration substrates [7–9]. Despite many achievements in the field of cell migration using these models, the resulting improved understanding of glioma migration [4] has had only a minor impact on therapy. These models have revealed that Rho-associated coil kinases (ROCKs) 1 and 2 (ROCK1 and ROCK2, respectively) are important molecules controlling glioma migration that act downstream of the small GTPase RhoA and functions by phosphorylating proteins such as myosin II, focal adhesion kinase, LIM kinase, myosin light chain phosphatase, and myosin light chain kinase. These various ROCK-mediated phosphorylation events organize the actin–myosin cytoskeleton [9, 10]. ROCKS are important regulators of microfilament structure, and they have been reported to both inhibit and promote cell migration in different cancer cells and in various migration assays [10–12]. In a brain slice assay, the inhibition of ROCK by fasudil immobilize glioma cells in intracranial xenograft models, preventing glioma progression [13]. Thus, the migration modes of gliomas vary within the complex brain environment, with some being ROCK dependent and others being ROCK independent. Live cell imaging of different substrates remains a challenge regarding the trade-off between in vivo complexity and spatiotemporal resolution of cell migration.

To generate such a true decision-taking situation for cells facing and crossing the interface between two substrates, we investigated migration preferences using a modification of the alternating stripe assay developed in the Bonhoeffer laboratory for studying axonal guidance (henceforth referred to as the Bonhoeffer assay) to study glioma cell migration [14, 15]. This device presents two alternating substrates to the cultured cells. We used five different substrates that were tested in pairs in every possible combination.

## Materials and Methods

### Cell Culture

Human glioblastoma cell lines U87MG, A172, D54MG, and 86HG39 (all cell lines are kind gifts of V. Senner, Institute of Neuropathology, Muenster, Germany) were cultured in Dulbecco’s modified Eagle’s minimal essential medium (DMEM) containing 10 % fetal calf serum, 100 U/mL penicillin, and 100 μg/mL streptomycin sulfate at 37 °C in an incubator with a 5 % CO_2_ atmosphere [all cell culture reagents were purchased from PAA (Linz, Austria)].

### Stripe Assay

**Fig. 1:**
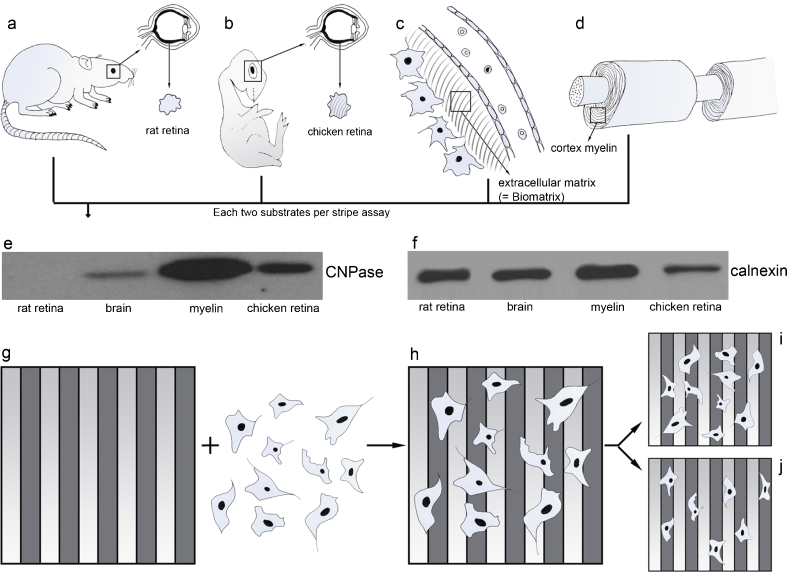
Schematic diagrams showing the experimental setup for creating alternating membrane stripes from different tissues and coculturing with U87MG cells. *a* Rat; *b* chick; *c* extracellular matrix (Biomatrix); *d* purified myelin from rat cortex; *e* immunoblot to test the content of myelin using CNPase in different substrates (rat retina, rat brain, chicken retina, and purified myelin) used for the stipe assay; *f* corresponding housekeeping control calnexin; *g* stripe assay setup with cells; *h* coculture of stripe assay with cells; *i* depiction of a stochastic preference in migration with a 50:50 distribution; *j* depiction of a 90:10 preferential distribution of cells

To create substrates that mimic the CNS environment, nonmyelinated neuronal membrane fractions were prepared from rat retina (Rr), myelinated membranes were derived from rat cortex (Rc), CNS myelin (M) was isolated from the postnatal rat cortex, and the ECM was a commercially available Biomatrix (BM). A total of 200 female pubs of the strain Sprague–Dawley were used for substrate preparation. The striped carpet comprised nucleopore filter membranes loaded in an alternating fashion with two substrates. The final stripe assay, consisting of two substrates and glioma cells, was then kept in an incubator at 37 °C for 24 or 48 h. During this period, the cells proliferated and migrated over the membrane carpets in the absence of mechanical barriers between the stripes. After 48 h, the stripe assays were evaluated to determine the cell preferences for either substrate. The profile of a stochastic cell distribution is shown in Fig. 1i, whereas Fig. 1j depicts a 90:10 hypothetical preference for one of the substrates tested (Fig. 1). The role of ROCKs was examined by treating the cells with the selective ROCK inhibitor Y27632, which was expected to influence substrate-dependent cell migration [16].

As stripe components, we chose homogenized rat retina to represent CNS tissue, which is easy to isolate and free of myelin, and oligodendrocytes to represent gray matter. For white matter, we chose embryonic chick retina, which, in contrast to rat retina, contains myelin (Fig. 1e, f). To reveal whether purified myelin has effects which differ from those of homogenized white or gray matter, we also isolated myelin from perinatal rat brains (Fig. 1e, f). For extracellular matrix, we chose the commercially available Biomatrix (Serva). The retinas of perinatal rats (postnatal day 10) and White Leghorn chicken embryos (embryonic day 10) were explanted and collected into ice-cold homogenization buffer (HB) containing a protease inhibitor cocktail (Complete EDTA free; Roche, Basel, Switzerland) (HB+). The tissue was homogenized first by titration through a 1,000-μL pipette tip and then through a G27× 0.75-in. needle. The cell membranes were separated using sucrose gradient centrifugation; 150 μL of a 5 % (*v*/*v*) sucrose solution was pipetted on top of 350 μL of a 50 % (*w*/*w*) sucrose solution and finalized with 800 μL of tissue homogenate. These setups were centrifuged at 5,525 rcf for 7 min at 4 °C, and the white rings between the sucrose layers were collected in 1,000 μL of precooled phosphate-buffered saline (PBS; pH 7.4) containing the protease inhibitor cocktail (henceforth referred to as PBS+). In a second centrifugation (5,525 rcf for 7 min at 4 °C), the remaining sucrose was washed out, excess liquid was removed, and the pellets were resuspended in 1,000 μL of PBS+ and stored in liquid N_2_. Myelin fragments were prepared from white matter tracts obtained from rat brains at postnatal day 10 as described above. Biomatrix EHS solution, a compound containing basement membrane components such as laminin, type IV collagen, entactin, and heparan sulfate, was also used.

The principle of the stripe assay is to present two alternating stripes of different substrates with no intervening mechanical barriers. This means that cells can freely choose between the different surfaces during migration, thereby revealing differences in affinity, motility, and cell proliferation. The aforementioned substrates were sucked onto a filter membrane (pore size 0.1 μm; Nucleopore track-etched polycarbonate membrane, Whatman, Maidstone, UK) via vacuum in a two-step process. The first set of stripes was prepared by sucking the suspension through a striped silicone matrix containing 90-μm-wide microchannels, upon which the filter membrane was mounted. The first stripes were formed in the area above the channels, while the intermediate spaces remained free. The second set of stripes was then prepared by changing the striped silicone matrix to a nylon grid, through which the suspension could pass unhindered. By then reapplying the vacuum, the second type of substrate settled within the unoccupied spaces between the first stripes, forming the second stripes. The filter membranes were rinsed in PBS, transferred to Petri dishes, and stored in PBS. Our stripe assay is a modification of the original assay of Bonhoeffer.

After trypsinization and counting the glioma cells, cell suspension containing cells at a density of 8 × 10^4^ cells/mL in DMEM (containing 10 % fetal calf serum, 100 U/mL penicillin, and 100 μg/mL streptomycin sulfate) was prepared, and 750 μL (= 6 × 10^4^ cells) of the suspension was added to the stripes. After the cells adhered to the membrane, they were allowed to migrate for either 24 or 48 h, after which the stripe assay cultures were fixed in 4 % paraformaldehyde and evaluated by means of immunocytochemistry. The effects of ROCK inhibition were examined by treating cells growing on either stripe with the ROCK inhibitor Y27632 (Sigma-Aldrich, St. Louis, MI, USA). Y27632 was dissolved in H_2_O to make a stock 3 mM solution. Y27632 has a half-life of 12–16 h [16], and thus 25 μL of the stock solution was added to the stripe assay cultures every 12 h to achieve an active final concentration of 100 μM, which has been shown to be the most effective concentration over 48 h. To distinguish between the two stripes being tested, one of them (usually the first) was labeled by immunocytochemistry. The glioma cells were also disclosed by immunostaining.

### Immunocytochemistry

Immunocytochemistry was performed as follows: the assays were incubated with fetal calf serum (FCS) containing 0.25 % Triton X-1000 for 2 h to prevent nonspecific antibody binding. After washing with PBS once, the first antibody (diluted in 10 % FCS) was added and incubated overnight at 4 °C. The assays were washed twice with PBS for 20 min each, and then the second antibody (also diluted in 10 % FCS) was applied and incubated overnight at 4 °C. The assays were then rinsed three times in PBS, transferred onto glass slides, and cover slipped with Mowiol (Hoechst, Frankfurt am Main, Germany) containing 4′,6-diamidino-2-phenylindole (Sigma-Aldrich) for nuclear staining.

The following antibodies were used to stain the stripes or glioma cells:

1. Biomatrix: anti-laminin IgG antibody developed in mouse (Sigma-Aldrich).
2. Rat retina: anti-rhodopsin IgG antibody developed in mouse (Millipore).
3. Chick retina: Anti-MOSP IgM antibody developed in mouse (Chemicon).
4. Myelin: anti-glial fibrillary acidic protein IgG antibody developed in mouse (Sigma-Aldrich).
5. Glioma cells: anti-vimentin IgG antibody developed in goat (Millipore).

The following secondary antibodies were used:

1. Tetramethyl-rhodamine isothiocyanate-conjugated anti-goat IgG antibody developed in donkey (Sigma-Aldrich).
2. Cy2-conjugated anti-mouse IgG antibody developed in goat (Jackson Immuno Research Laboratories, Suffolk, UK).
3. Fluorescein isothiocyanate-conjugated anti-mouse IgM antibody developed in goat (Jackson Immuno Research Laboratories).

Immunofluorescence was visualized using a fluorescence microscope (Axiophot, Zeiss, Oberkochen, Germany) attached to a CCD camera (Zeiss) and was documented digitally (AxioVision, Zeiss).

### Immunoblotting

Immunoblotting was performed as described previously [16]. Briefly, membranes were blocked using 5 % skimmed milk powder for 30 min and then incubated with a primary rabbit anti-ROCK1 antibody (diluted to 1:1,000; Sigma-Aldrich) or with a mouse anti-myelin CNPase antibody (diluted 1:1,000; Covance) overnight at 4 °C. Protein signaling was detected using a peroxidase-conjugated secondary anti-rabbit antibody (NA9340, Amersham) or a peroxidase-conjugated secondary anti-mouse antibody (A3682, Sigma) and Amersham electrochemiluminescence western blotting detection reagents (GE Healthcare). The membranes were reprobed with an antibody raised against calnexin (diluted to 1:2,000; Sigma-Aldrich) as a control for the amount of protein loaded.

### Analysis and Statistics

For cell preference analysis, images were blinded for stripe composition and evaluated by two investigators. There was no interobserver variability in the evaluation of the experiments. The percentages of cells on the different stripes were calculated. Statistical analysis was performed with SPSS 14 software (IBM). Results are presented as mean percentage values, and error bars represent SEM values. Statistical significance was analyzed using the paired Student’s *t* test, and asterisks indicate the representative level of probability (i.e., *p < 0.05; **p = 0.001; ***p < 0.001).

### Atomic Force Microscopy

Atomic force microscopy (AFM) was performed on an Autoprobe CP atomic force microscope from Park Scientific Instruments equipped with a laser beam bounce detection system and a piezo tube sample scanner for the movements in the lateral and vertical directions. The AFM measurements were conducted in the operation mode of amplitude-modulated intermittent contact under ambient conditions in air. The working procedure for AFM measurements was to image a certain surface area (80 × 80 μm) of the stripes and to shift the scan area with respect to the subsequent image by about 70 μm to ensure complete coverage of a larger investigation area. A marker was attached mechanically to the sample surface structure to allow the investigated areas to be identified in the subsequent fluorescence microscopy.

### Quantitative Real-Time PCR

Quantitative RT-PCR was performed by isolating RNA from human glioma cell lines using the GenElute Mammalian Total RNA Miniprep kit (Sigma-Aldrich), reverse transcription of total RNA using a High-Capacity cDNA reverse transcription kit (Applied Biosystems), and amplifying cDNA on a StepOne Plus sequence detection system (Applied Biosystems) using the following primers specific for the respective transcripts in duplicates: ROCK1 human (forward, AAAAATGGACAACCTGCTGC; reverse, GGCAGGAAAATCCAAATCAT) and ROCK2 human (forward, CGCTGTCCGAGACCCT; reverse, TTGTTTTTCCTCAAAGCAGGA). Data were normalized for glyceraldehyde-3-phosphate dehydrogenase expression using the comparative threshold cycle (ΔCt) method.

### Pathway Analyses

For signaling pathway analyses, human glioblastoma cells D54MG and 86HG39 were cultured on coated flask for 72 h in a density of 1 × 10^6^ cells. Three different coatings were used: Biomatrix as an example for ECM, myelin from rat brain as an example for gray matter, and retina from rat as an example for unmyelinated tissue (white matter).

After cells grown for 72 h on the different surfaces, cells were removed and prepared for western blot analysis. Lysis of cells and tissue, SDS-PAGE, and blotting were done as described previously [16]. The rabbit anti-ROCK1 antibody (Sigma-Aldrich, 1:2,000), diluted 1:1,000, rabbit anti-ROCK2 antibody (Sigma-Aldrich, 1:2,000), anti-RhoA (Abcam, mouse, 1:500), anti-pLIMK (cell signaling, rabbit, 1:1,000), anti-pMLC, and anti-pCdc42/Rac (cell signaling, mouse, and rabbit, respectively, 1:1,000) were incubated overnight at 4 °C. As secondary antibodies, a peroxidase-conjugated goat anti-rabbit antibody (Sigma A9169) was used at 1:50,000 dilution, and a goat anti-mouse antibody (Sigma, A3682), at 1:50.000 dilution and incubated for 1 h at room temperature. To verify equal protein loading on each lane, blots were stripped and reprobed for rabbit anti-Calnexin (Sigma-Aldrich, 1:2,000).

## Results

**Fig. 2:**
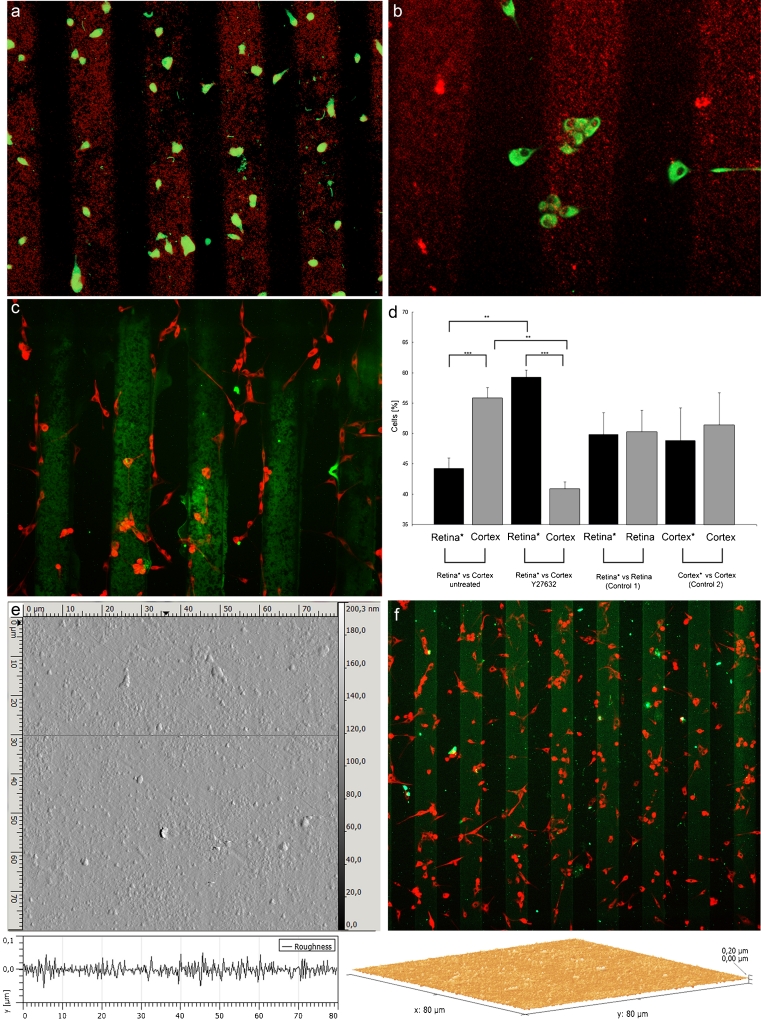
Example of cell growth on nonmyelinated rat retina vs myelinated rat cortex substrate. **a** Demonstration of a membrane with cells preferring cortex (*red*) as migration substrate. **b** Larger magnification of a parallel experiment showing individual cells that extend growth processes and prefer cortex (*red*) over retina as substrate. **c** Addition of Rho kinase inhibitor Y27632 allows growth of the cells, but with reversal of the preference. **d** Quantitative analysis showing that the inhibitor of Rho-associated coil kinase reverses the preference of glioma cells for cortex as migration substrate. Repeating the setup with retina/retina or cortex/cortex showed no preference. **e** Atomic force microscopy showing the surface of a stripe assay with no detectable difference in height between the stripes. **f** Example of a stripe assay showing uniform distribution of cells across the membrane

In the first set of experiments, we tested whether the gliomas exhibited a preference between myelinated and nonmyelinated tissue and cultured the cells on stripes loaded in an alternating fashion with either rat cortical or retinal tissue. It appeared that most of the cells preferred to grow on cortical tissue (at a ratio of about 60:40; Fig. 2a, d). Most of the cells were aligned along the substrate interface, mostly preferring to grow on cortical membranes (Fig. 2b). The preference between cortex- and retina-derived membranes was significantly reversed when the ROCK inhibitor Y27632 was added to the cultured assays (to a ratio of about 20:80; Fig. 2c, d). Figure 1d shows the counts of cells growing on retina/cortex stripes. No reversal was observed in controls using retina/retina or cortex/cortex stripes, and the preference remained at about 50:50 (Fig. 2d). Atomic force microscopy images showed a uniform plateau of the stripe channels loaded with membranes (Fig. 2e) and a uniform distribution of cells (Fig. 2f), indicating the absence of physical irregularities at the stripe interface. Therefore, the data demonstrate that the preferential distribution of cells on the stripes is unlikely to have been caused by differences in membrane altitude.

**Fig. 3:**
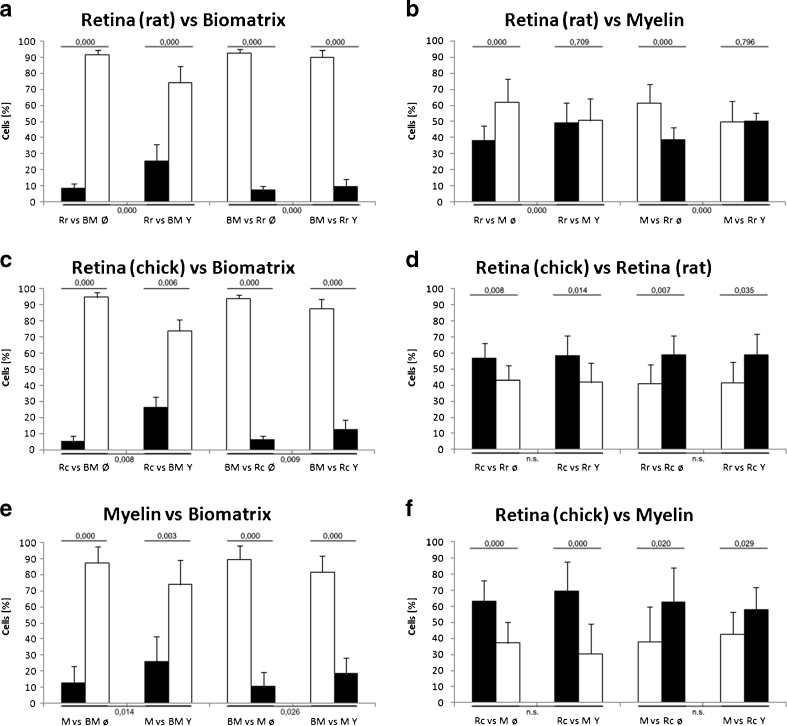
Histograms showing the quantification of U87MG cells growing on alternating stripes. In all boxes, two setups are shown with a reversed arrangement of membranes on the stripes. For instance, retina first and BM second (*left*) and then BM first and retina second (*right*) during the production of stripes to ensure that differences are not attributable to the stripe production process. **a** It appeared that BM without inhibitor (i.e., Rr vs BM Ø and Rr vs BM Ø) attracted most of the cells when confronted with rat retina, whereas treatment with the inhibitor was effective (i.e., Rr vs BM Y and BM vs Rr) and significantly reduced the preference for BM. **b** Confronting Rr with purified rat cortex M revealed a significant preference for M. This preference was neutralized after treatment with the inhibitor. **c**–**f**) Further confrontations of substrates showing clear preferences for BM over chick retina (**c**), Rc over Rr (**d**), M over BM (**e**), and Rc over M (**f**). *Horizontal bars* over each arrangement show the statistical probability values

The cell preferences for myelin, white matter, gray matter, and ECM in vitro were determined by presenting the substrates against each other with the stripe assay. The differences in cell preferences were greatest between stripe composites of neuronal tissue and ECM. When gray matter (e.g., Rr) was presented vs BM, 91.6 % (in the Rr vs BM Ø setup) and 92.6 % (BM vs Rr Ø) of the cells resided with the BM stripes, respectively (Fig. 3a). With ROCK inhibition, there was a shift toward retina tissue with 25.7 % (Rr vs BM Y) and 9.8 % (BM vs Rr) of glioma cells settling on the Rr stripes, respectively (Fig. 3a). When Rr was presented vs cortical M (Rr vs M Ø and M vs Rr Ø), significant preferences for M of 60.9 and 61.6 % were found in both setups, respectively (Fig. 3b). With ROCK inhibition, the preference was neutralized, with preferences of 50.6 and 49.6 %, respectively (Fig. 3b). Presenting white matter (e.g., Rc) vs BM produced approximately the same proportions of cells settling, with 94.6 % (Rc vs BM Ø) and 93.7 % (BM vs Rc Ø) of cells preferring BM (Fig. 3c).

With ROCK inhibition, significantly more cells adhered to the retina substrate in both setups (Rc vs BM Y, 74.0:26 %; BM vs RC Y, 87.6:12.4 %; Fig. 3c). When we presented white matter (i.e., Rc) vs gray matter (i.e., Rr), we observed a distinct preference for Rc (Rc vs Rr Ø and Rr vs Rc Ø), but no shift in allocation with ROCK inhibition in both the Rc vs Rr Y and Rr vs Rc Y setups (Fig. 3d). In assays in which M was set against BM, the preferences were 87.3 to 12.7 % (M vs BM Ø) and 83.4 to 16.6 % (BM vs M Ø) in favor of BM; the distributions changed slightly with the ROCK inhibitor Y27632, with 74.0 and 81.6 % preferring BM, respectively (Fig. 3e). When the Rc was presented vs M, there was a significant preference for cortical M in both setups (Fig. 3f). This preference was not blocked by addition of the inhibitor (Fig. 3f). The data show that most U87MG cells preferentially grew on ECM followed by cortical M and extracerebral nervous tissues such as myelinated Rc and nonmyelinated Rr. Some of these preferences were neutralized by the ROCK inhibitor.

**Fig. 4:**
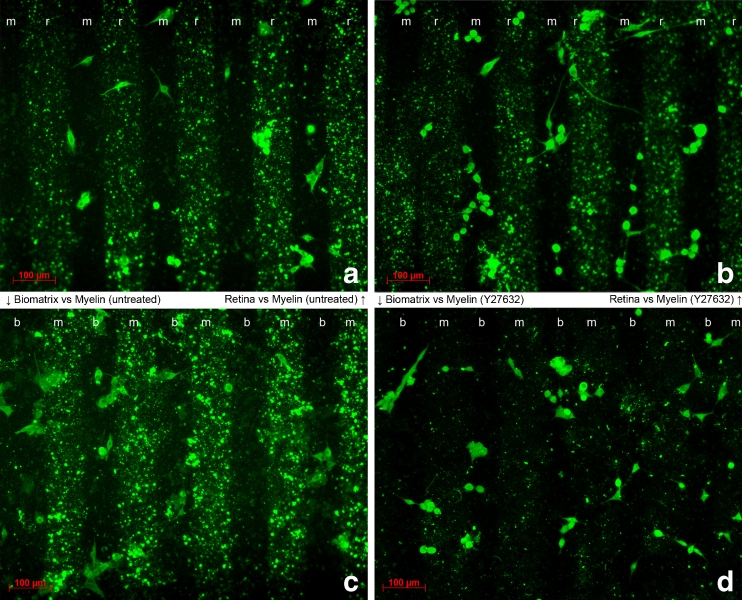
Fluorescence photomicrographs showing the distribution of U87MG cells on alternating stripes: **a** Rr vs M without inhibitor, **b** Rr vs M plus ROCK inhibitor, **c** BM vs M without inhibitor, and **d** BM vs M with inhibitor. *Scale bar*, 100 μm

The physical location of the glioma cells on either stripe was determined at the end of each experiment using selected antibodies that facilitated recognition of the stripes and cells. Figure 4 shows representative fluorescence images used to quantify the data. There was a clear preference of the cells for M over Rr in the untreated setup. This preference was partly neutralized in inhibitor-treated setups. When BM was tested vs M, most of the cells adhered to the BM. This strong preference was also neutralized when the inhibitor was added to the stripes (Fig. 4).

**Fig. 5:**
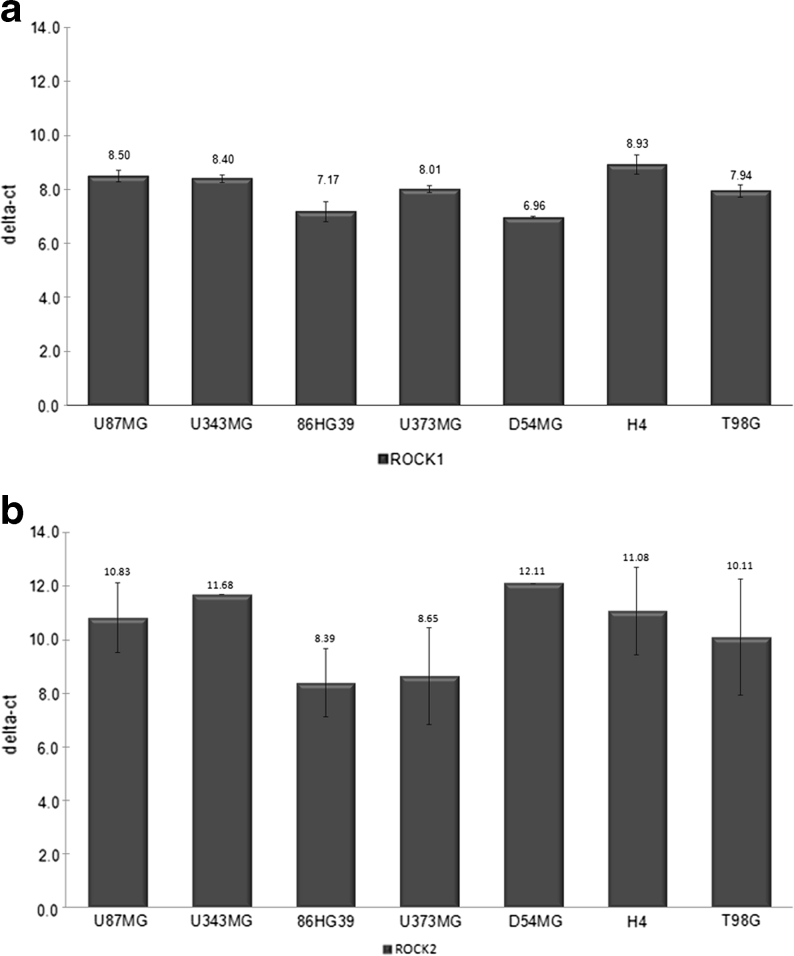
mRNA expression of ROCKs in glioblastomas. **a** Quantitative RT-PCR of ROCK1 mRNA in different glioblastoma cell lines revealing comparable ΔCt values of seven different cell lines. **b** mRNA expression of ROCK2 mRNA in different glioblastoma cell lines

**Fig. 6:**
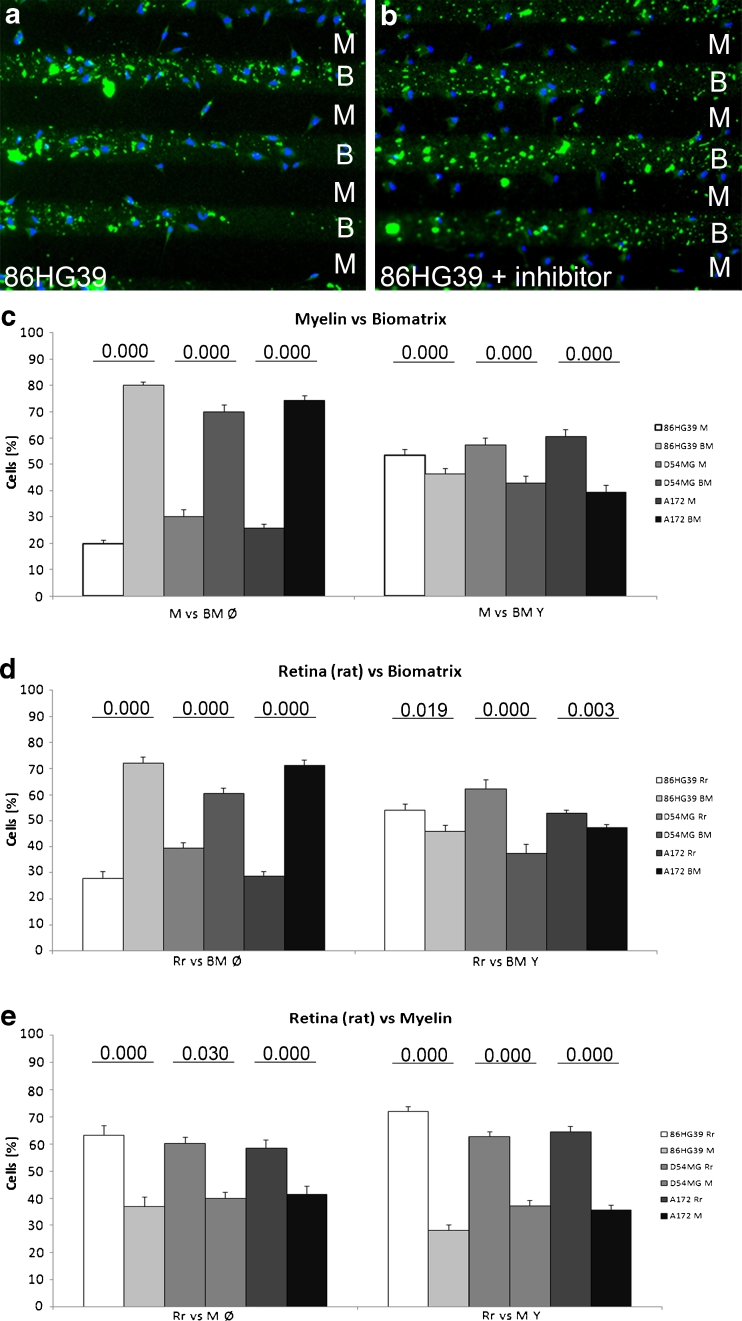
Substrate preferences of different glioblastoma cell lines. **a**, **b** Distributions of 86HG39 cells cultured on the stripe assay consisting of myelin (*M*) or Biomatrix (*BM*) without (**a**) and with (**b**) the inhibitor. Note that the inhibitor results in a substantial redistribution of cells from BM to M. **c**–**e** Quantification of three cell lines tested in the stripe assay without (*left histograms*, Ø) and with (*right histograms*, Y) the inhibitor

To examine whether the results obtained with the human glioblastoma cell line U87MG also applied to other cell lines, we cultured three other cell lines (86HG39, D54MG, and A172 cells) with the stripe assays composed of matrix, rat M, and nonmyelinated Rr and tested them against each other. It appeared that all of these cell lines expressed ROCK1 and ROCK2 mRNA at comparable levels (Fig. 5). In immunoblots, ROCK1 and ROCK2 were expressed at protein levels (data not shown). As shown paradigmatically in Fig. 6a, b for the 86HG39 cell line, the cells clearly preferred BM over M (Fig. 6a), and this preference was changed after inhibition of ROCK1 (Fig. 6b). This change in the substrate preference was shown quantitatively in stripes testing either M vs BM in three cell lines (Fig. 6c) or Rr vs BM (Fig. 6d). In the testing of M vs Rr, there was a preference for retinal membranes over M (Fig. 6e), with the addition of the inhibitor further enhancing this preference in all three cell lines tested (Fig. 6e). These data indicate that the stripe assay is a suitable and sensitive method for detecting ROCK1-dependent changes in the preference of glioblastoma cells.

**Fig. 7:**
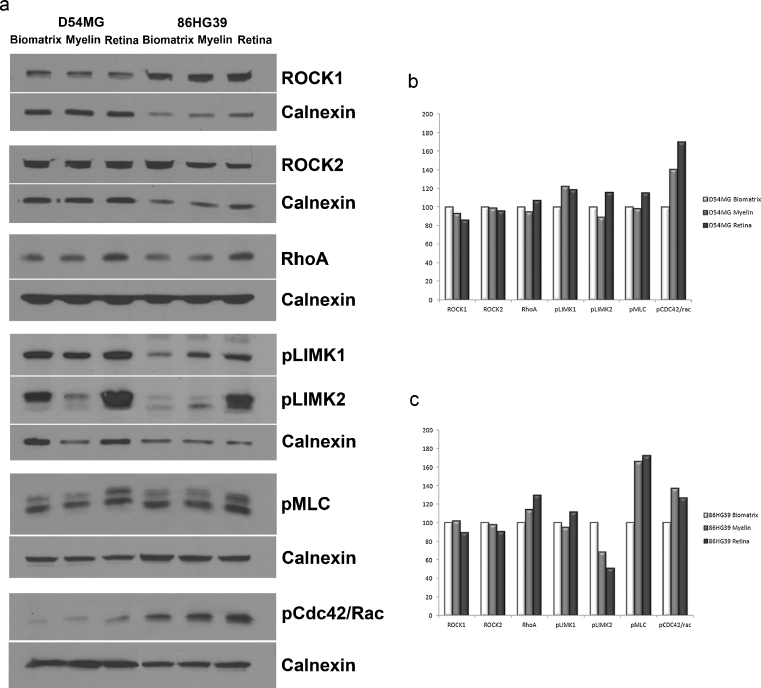
Signaling pathway analyses of glioblastoma cell lines growing on three different substrates. **a** Western blot analyses of the cell lines D54MG and 86HG39 growing on Biomatrix, myelinated brain tissue, and unmyelinated tissue from rat retina. ROCK1, ROCK2, RhoA, phosphoLIMK1, phosphoLIMK2, phosphoMLC and phosphoCdc42/Rac protein expression were analyzed. **b**, **c** Densitometric measurement of western blots for D54MG cells (**b**) and 86HG39 cells (**c**). Cells grown on BM displayed a higher expression of ROCK1 and ROCK2 compared to cells growing on myelin and retina. Furthermore expression of all tested proteins varies between the three substrates cells grown on

**Fig. 8:**
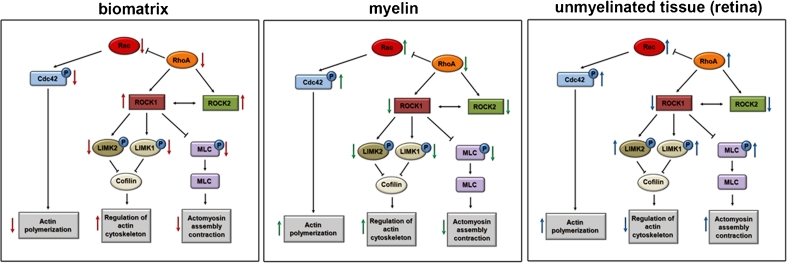
Graphs showing the different regulation of glioma cell signaling when cells were cultured on biomatrix, myelin and unmyelinated tissue (retina). *Arrows* indicate the up- and downregulation of intracellular signaling members of the Rho kinase pathway. These differences likely explain that alternative pathways exist for migration on different substrates

To examine possible changes in intracellular signaling pathways dependent on cell growth on different substrates (biomatrix, myelin, and retina), western blotting was used with the lines DM54MG and 86HG39 which expressed variable, yet comparable amounts of ROCK1 and ROCK2 (Fig. 7). Expression of RhoA was enhanced in cells on unmyelinated tissue. P-LIMK1 revealed a different expression level in both used cell lines. For D54MG, expression of p-LIMK1 was increased on myelinated surfaces, for 86HG39 on unmyelinated surfaces. P-LIMK2 displayed also a cell-based expression level with the highest expression on unmyelinated tissue for D54MG cells and on Biomatrix for 86HG39 cells. P-MLC showed an increased expression on unmyelinated tissue in both cell lines. pCDC42/Rac displayed an elevated protein expression level in cells grown on unmyelinated tissue compared to Biomatrix coating (Fig. 7). Figure 8 illustrates the cell signaling pathways on the different substrates.

Cell motility studies were essential for monitoring how the cells gained their final distribution on the alternate stripes. Representative sequences of the assays over time were recorded using time-lapse videomicroscopy. This demonstrated clearly that untreated U87MG cells meandered around the substrates and selectively preferred to attach and grow on BM over M (link to video 1 and legend “untreated” in the link http://www.melkonyan.de/ROCK/ROCK.php). Inhibition of ROCK with Y27632 attenuated the cell motility and diminished their selectivity for either substrate (link to video 2 and legend “treated with ROCK inhibitor” in the link http://www.melkonyan.de/ROCK/ROCK.php).

## Discussion

It is highly likely that the direction of glioblastoma migration is not determined by a single mechanism, but rather occurs via different pathways depending on the microenvironment that is serving as a substrate. The advantage of the stripe assay over conventional models used to study cell migration is the opportunity to present two alternating substrates and to examine the preference for either of them in a controlled fashion. The data presented here support the view that techniques used to unravel the mechanisms of cell migration within the complex brain environment may involve the selective inhibition of these cells and blockage of diffuse migration within the tissue. However, more substrates, such as components of the brain vessels or individual constituents of the ECM, need to be tested. Although human cell lines were tested, they showed a clear preference on rat myelin or on rat and chicken retinal membranes. These data point to cross-reactivity between the species. However, rat cell line C6 has been recently tested by using chick neuronal fibers—C6 assay—and showed similar properties in relation to expression of ROCK and cell migration [16].

The high resolution of the stripe assay revealed that ROCK inhibition resulted in changes in cell preferences, and especially in a shift away from ECM toward each other brain substrate. From the therapeutic point of view, these changes are fundamental in order to understand that glioma cells may use alternative pathways of migration, likely by using different cell signaling. In turn, knowledge of these different signaling pathways may help to better understand the molecular mechanisms of migration, and thus of designing therapeutic strategies. These findings are especially interesting when combined with the previous finding that glioblastoma cell migration on ECM is not ROCK dependent [10–12, 17]. The increased cell preference for white matter, gray matter, and M when presented vs ECM indicates an immobilization of glioblastoma cells due to ROCK on all the neuronal substrates: white matter, gray matter, and purified M. When only neuronal substrates (white matter, gray matter, and M) were used in the ROCK inhibition assays, there was a trend for cells to be more profoundly inhibited on white and gray matter than on M, indicating that ROCK is required more for migration along axonal structures, whether myelinated or nonmyelinated, than on purified M. ROCK seems to be a major molecular player required for the migration of glioblastoma cells along the tissues of their preferred migration routes in vivo: axons, myelinated axons, and purified M. As shown recently, cytokine signaling through the receptor GP-130-IL6ST and the kinase JAK1 also generates contractility of cellular actomyosin through ROCK-dependent signaling [12]. It is therefore not surprising that ROCK inhibition has been shown to suppress glioblastoma growth in vivo [13, 16].

In summary, the findings of this study demonstrate clearly that glioblastoma migration and substrate preferences are distinct to the cellular and acellular substrate, which helps to explain the great complexity of tumor migration within the brain. In this context, the stripe assay may be able to reveal the molecular cues of glioblastoma migration on each of the substrates that are used in vivo, as predicted from its original use. The stripe assay setup represents a compromise between the complexity of in vivo models and the high resolution of in vitro models that is necessary to reveal fundamental cellular processes, and can be successfully transferred to tumor research. It is likely that the stripe assay will also be applicable to other tumors throughout the body.
